# Combined driving: task-specific position impacts grip strength of equestrian athletes

**DOI:** 10.1186/s11556-021-00282-w

**Published:** 2022-01-10

**Authors:** Michaela M. Keener, Kimberly I. Tumlin, Nicholas R. Heebner

**Affiliations:** 1 Sports Medicine Research Institute, Department of Athletic Training and Clinical Nutrition, College of Health Sciences, University of Kentucky, 720 Sports Center Drive, Lexington, KY 40506 USA; 2 Department of Epidemiology, College of Public Health, University of Kentucky, 720 Sports Center Drive, Lexington, KY 40506 USA

**Keywords:** Equestrian athlete, Handgrip strength, Combined driving, Sport performance, Aging adults

## Abstract

**Background:**

Loss of hand strength is a predictor of mortality in aging populations. Despite reliance on the hands to participate in equestrian driving activity, no existing studies focus on associations of hand strength to athletic performance. Therefore, this study 1) established baseline handgrip of equestrian combined drivers in standing and task-specific positions, 2) determined endurance of task-specific handgrip, 3) compared handgrip strength to normative data, and 4) evaluated associations of handgrip and equestrian-specific variables.

**Methods:**

There were 51 combined drivers (9 males, 42 females) ages 21–78 who completed a survey, standing handgrip, and grip strength and endurance in a task-specific position. Sixty-three percent of participants were 50 years or older. The dynamometer grip bar was normalized by hand size for standing tests; to duplicate sport-specific tasks, the bar was set to the closest setting. Significances were determined at *p* < 0.05.

**Results:**

Drivers with more than 30 years of experience demonstrated highest summed standing (73.1 ± 5.2 kg) and summed sitting (59.9 ± 6.3 kg) grip strength. Females 60-years and older had greater handgrip endurance (Χ^2^ = 8.323, df = 2, *p* = .0156) in non-dominant (left) hands. Males (60%) reported more cold weather fatigue than females. Glove wearing was associated with bilateral endurance balance; a higher proportion of endurance balance between dominant and non-dominant (49% high-high and 29% low-low; Χ^2^ = 11.047, df = 1, *p* = .0009) was realized. There were no associations of handgrip and prior injury.

**Conclusions:**

Our results have implications in understanding task-specific and normative grip strengths in aging equestrian populations. Bilateral balance in handgrip strength and endurance is important particularly in maintaining strength in non-dominant hands over time. Equestrian driving sport promotes greater endurance in older females. Strength can be improved by participating in combined driving, and engagement in this sport over several years’ benefits hand strength over time. This cohort of equestrian participants provides evidence that participating in hand-specific activities promotes greater strength, which has been previously shown to improve aging outcomes.

**Supplementary Information:**

The online version contains supplementary material available at 10.1186/s11556-021-00282-w.

## Background

Handgrip strength (HGS) outcomes have been associated with health risks in aging populations. Generally handgrip strength increases during childhood and peaks in early adulthood. A pattern of handgrip decline is predictive of aging outcomes [1, 2]. Mortality is increased when HGS is low and general weakness limits daily functioning [1, 3]. Likewise, handgrip endurance (HGE) is necessary and more prevalent for completing daily tasks than maximal grip strength [4]. Decreased HGS has been linked to disability and decreased ability to complete daily activities; whereas increased HGS later in life is linked to higher ratings of quality of life (QoL) [5, 6]. Measuring HGS can indicate sarcopenia, as grip force is an indicator of physiological functioning in the hands and upper limbs [3, 7]. When compared to younger individuals, elderly adults who completed grip force measurements using a hand dynamometer has slower and inconsistent maximum grip values, suggesting the need to target activities which train both speed and consistent grip in aging adults [3, 8–10]. Research has shown individuals participating in activities that require constant use of the hands, such as tennis or rock climbing, have greater HGS and HGE than non-active individuals [8–11]. However, to date no research has evaluated and reported HGS and HGE of equestrian sport despite reliance on HGS to communicate and navigate during these specialized activities.

Equestrian sport is the only physical activity where individuals of all ages and sexes compete equally across the lifespan. Older adults can participate internationally for competition in combined driving or recreationally in equestrian carriage driving without being mounted on the horse. In the United States, over 90% of participants in equine activities are female although in Thoroughbred racing males dominate the sport [12]. This split leads to research gaps in understanding sex differences as it relates to specific equestrian disciplines with more than 4.6 million Americans actively involved in equestrian sport or industry and an estimated 20 million riders in developed countries [12, 13]. Similar to other sports, each discipline relies on unique skill sets. Juxtaposing physical demands on musculoskeletal health while considering differences between the sexes and among age groups would inform equestrian health, safety equipment, education, and policies.

Combined driving is a specific discipline within equestrian sports. Combined Driving Events (CDE) are comparable to the human triathlons, testing the driver’s ability to navigate their horse(s) and carriage through event disciplines: dressage, marathon, and cones. Combined drivers are among the few equestrian athletes whose central means of communicating with their horse(s) relies on their hands put precise pressures on the reins and bit. In contrast, mounted riders communicate with their horse using multiple natural cues, such as leg movement and overall posture, in addition to their hands. Drivers guide one, two (“pair”) or four (“four-in-hand”) horses at a time in three phases. As competition level increases, so does the difficulty of control and physical demands. The team must have synchronism for the dressage phase, stamina for the marathon phase, and agility for the cones phase with all communication initiated and processed through the driver’s hands. These events occur over multiple days and drivers are actively participating a minimum of one hour daily. Due to the reliance on hand function in this discipline, understanding how hand function may promote healthy aging is vital to advancing health and safety in these populations.

Equestrians also provide care for their horses. Hand movements range from small intrinsic half-halts (e.g. squeezing on the driving lines) to more extensive extrinsic muscle activation, including carrying water buckets and mucking stalls. Previous research indicated that horseback riders had average grip strength values respective to their sex and age [14, 15]; however, these studies did not address aging populations nor driving activity. Additional determinants affecting grip strength such as such as activity experience, injury, number of horses in a team, and the percent of barn activities completed by the drivers has not been evaluated. Measuring these values is important in determining if similar combined driving helps promote hand strength as demonstrated in other sports [5, 8–10]. Drivers use their hands continuously and engage in equine-specific variables. Given these activities, we hypothesized that combined drivers’ handgrip strength would be above average normative data based on their respective sex and age range. The purpose of our study was fourfold: 1) to establish baseline data on grip strength of combined drivers in standing and a task-specific position; 2) to create baseline data on grip endurance in combined drivers in a task-specific position; 3) to compare grip values to normative data by driver age and sex; 4) to determine associations between equestrian-specific and grip variables.

## Methods

We used a single-sample, cross-sectional analysis to establish normative HGS in a novel population of equestrian combined drivers. HGS was evaluated in two positions: a handgrip position typically used in clinical settings and a task-specific position. A questionnaire was administered to all participants to define equestrian-specific grip variables including: injury history; hand-associated conditions (i.e., arthritis); driving experience; number of horses in their team; and engagement in external activities related to the sport (daily horse care and barn activities). A small panel of combined drivers reviewed survey questions and the questionnaire was edited for relevancy and clarity based on their input.

Fifty-one combined drivers (9 males and 42 females) ages 21–78 were recruited during two national-level CDEs during August and September 2019. All drivers were right-hand dominant, and 63% were age 50 years or older. Participants were included if they were currently competing or had competed in a CDE in the past year, were between ages 18–80 years, and were free of a current injury. Participants with arthritis were included in this study. All subjects signed an informed consent approved by *the University of Kentucky* Intuitional Review Board.

**Fig. 1:**
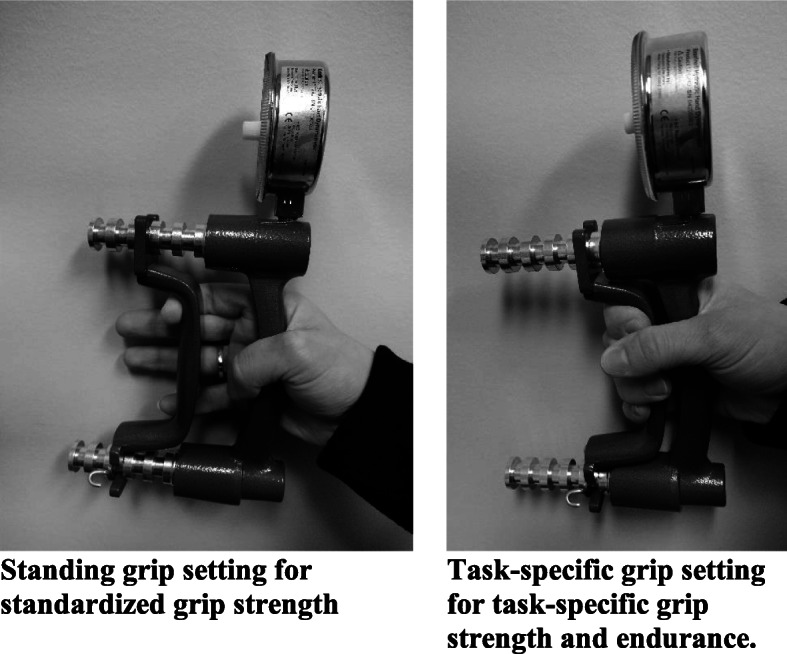
Grip setting for testing protocols

**Fig. 2:**
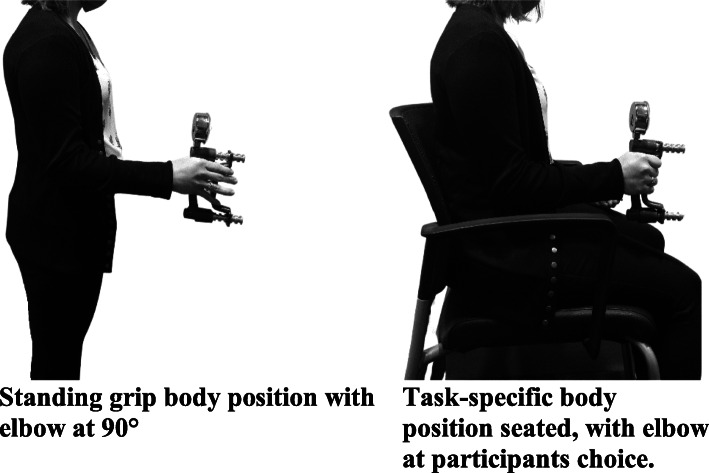
Positioning for Standing Grip Strength

Investigators were not blinded. To establish baseline data of hand strength comparatively to normative values, participants first completed three maximal voluntary control (MVC) squeezes on a manual hand dynamometer with both right and left hands. Participants stood upright, with feet hip-distance apart. Participants self-selected the order of the hand tested first; this remained the same throughout the remaining tests. Each hand was individually fitted to the hand dynamometer, so the grip size was consistent where the second phalanx of the middle finger was approximately even with the grip bar (Fig. 1). If a participant’s ideal hand position was between two positional settings, the grip bar was configured to the proximal setting. Participants were instructed to keep their elbows at ninety-degrees, close to their side, and keep their wrist straight, with their thumb upright as they completed an MVC while holding the adjusted dynamometer (Fig. 2). Investigators provided verbal encouragement to each participant while completing the MVC. Three MVC’s were recorded with minimal breaks between each attempt before switching to the other hand.

**Fig. 3:**
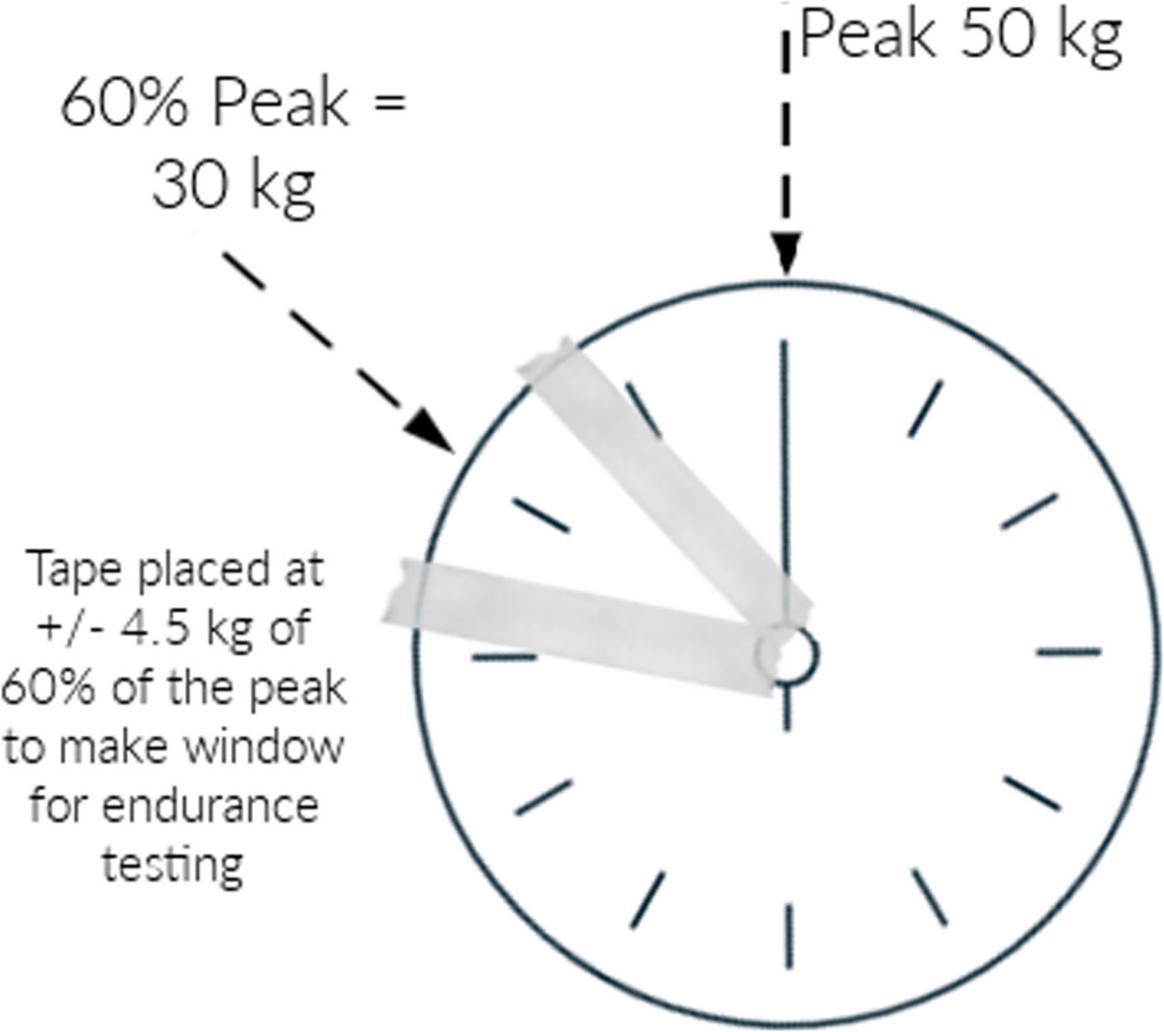
Hand dynamometer setting at 60% peak HGS

A task-specific position was created by setting the hand dynamometer to the most proximal setting (Fig. 1) to mimic driving reins to establish baseline data of combined drivers’ grip strength and endurance. Participants were asked to sit in a chair (Fig. 2), as they sit while driving. Three MVC’s in this task-specific position were recorded on each hand. Sixty percent of the achieved peak MVC on each hand in the task-specific position was calculated. Using masking tape, researchers created a visual window on the dynamometer analog gauge that marked a range on the gauge that was +/− 10 pounds of the calculated 60% peak MVC as shown in Fig. 3. Participants remained seated, faced a mirror, and were instructed to use the reflection of the dynamometer gauge in the mirror to maintain their grip such that the gauge needle remained between the two pieces of tape on the gauge for as long as possible. The researcher started a timer once the needle was in the marked range. A total time was recorded when the dynamometer needle fell below the lower mark on the dynamometer (e.g., the 50 lbs. mark in Fig. 3). Researchers stopped the participants if they reached a 2-min time of endurance based on prior feedback from combined drivers and pilot testing.

Descriptive statistics and frequencies were used to summarize the demographics of the driving population. Reported driving experience was coded to experience categories of a) 5 years or fewer, b) 6 to 15 years, c) 16–29 years, or d) 30 or more years. Number in hand refers to the number of horses that the driver controls at one time during the competition day: one, two, or four horses. Body mass index was grouped into underweight, normal, overweight, and obese. Relationships of driving experience, handedness, and normative HGS data were evaluated by sex and number in hand. Mean positional HGS (kg) and sitting HGE (kg) were calculated for each hand. Standing HGS was summed for both hands and coded into normative categories of those below the 20th, 20th–50th, 51-80th, and above 80th percentiles for previously reported grip strengths (in pounds) by age and sex [16]. These percentiles were converted to kilograms. We evaluated, age effects in each HGE category by sex. Ages were split into three groups based on frequencies within each age: under 49 years old, 50–60 years old, and above 60 years. Sitting HGE values were coded into binary categories of low (less than a minute) and high (more than a minute). HGE differences from dominant to non-dominant hand were calculated and used for comparisons to equestrian-specific variables. Report of prior injuries, use of gloves, type of fatigue, and amount of horse and barn activities completed by participants was paired with HGS and HGE data. Comparisons were made using Chi-square or ANOVA, as appropriate.

## Results

Participants were primarily Caucasian females with 6 to 15 years of driving experience and ranged in ages from 24 to 79 years (Table 1). The median age was 56 years, and all were right-hand dominant. Many drivers handled one horse in hand, with 12% of females driving four horses in hand. Of the male participants, 66% were overweight or obese, compared to 59% of the female drivers.

**Table 1:** Demographics of Combined Driving Event participants by sex

	Males (*n* = 9)	Females (*n* = 42)
Age	48.7 ± 13.7	52.8 ± 13.7
	(21–66 years)	(24–79 years)
Race	White/Caucasian (89%)	White/Caucasian (98%)
	American Indian/Alaska Native (11%)	American Indian/Alaska Native (2.5%)
Combined Driving Experience	5 or less - 33.33% (3)	5 or less years - 28.6% (12)
	6 to 15–22.225 (2)	6 to 15–40.5% (17)
	16 to 29–33.33% (3)	16 to 29–11.9% (5)
	More than 30 years - 11.11% (1)	More than 30 years - 19% (8)
Number of horses in hand	One (88%)	One (80%)
	Two (11%)	Two (7%)
	Four (0%)	Four (12%)
Self-Selected Dominant Hand	Right	Right
BMI Category	Under (0%)	Under (2%)
	Normal (33%)	Normal (39%)
	Overweight (44%)	Overweight (27%)
	Obese (22%)	Obese (32%)

Positional HGS (Table 2) varied by sex, with males having higher HGS (*p* < 0.0001) than females for both positions (standing and sitting), as well as bilaterally. Within females, positional HGS was higher in both hands (*p* < 0.0001) while standing. Males demonstrated higher (*p* = 0.0375) standing HGS only in the left hand. Collectively, years of driving experience numerically had the highest values for summed standing (73.1 ± 5.2 kg) and summed sitting (59.9 ± 6.3 kg) HGS for all drivers with more than 30 years of experience. Males with 16 to 29 years of experience had the highest mean HGS in the standing position (90.9 ± 8.5 kg). Age-related normative percentiles showed males have numerically lower HGS than females at ages below 30 years. There were no associations of sex and HGE by hand or in endurance difference between dominant and non-dominant hand.

**Table 2:** Positional hand grip strength (HGS), normative percentiles of HGS, and endurance (HGE) by sex

	Males (*n* = 9)	Females (*n* = 42)
Sitting HGS (kg)	Right Mean: 37.7 ± 7.9^a^	Right Mean: 24.5 ± 8.4^b^
	Left Mean: 35.7 ± 9.5^a^	Left Mean: 22.7 ± 8.2^b^
Summed HGS Sitting (kg)	73.4 ± 17.4^a^	47.0 ± 16.3^b^
Standing HGS (kg)	Right Mean: 43.3 ± 5.4^a^	Right Mean: 32.1 ± 6.6^b^
	Left Mean: 44.1 ± 7.3^a^	Left Mean: 30.4 ± 6.4^b^
Summed HGS Standing (kg)	87.4 ± 12.0^a^	62.5 ± 12.6^b^
Normative Percentile		
18–25 years	20th -50th	>80th
25–30 years	–	>80th
31–40 years	20th -50th	>80th
41–50 years	51st-80th	51st-80th
51–60 years	51st-80th	51st-80th
Over 60 years	20th -50th	51st-80th
Endurance	Right 78.7 ± 11.4 s	Right: 72.9 ± 5.3 s
	Left: 67.3 ± 11.4 s	Left: 63.3 ± 5.3 s
Endurance Difference (dominant to non-dominant hand)	11.3 ± 7.2 s	9.5 ± 3.4 s

^ab^Values with unlike superscripts differ at *p* < 0.0001 within position

HGE category was evaluated bilaterally and by sex for associations (Table 3). There were no associations between sex and bilateral endurance difference. Within females, HGE category was greatest (Χ^2^ = 8.323, df = 2, *p* = .0156) in left hands (non-dominant) in the above 60-year age group. This effect was not seen in males in either hand or seen in female right (dominant) hands by age group.

**Table 3:** Hand grip endurance (HGE) category associations with participant demographics

	Males (*n* = 9)	Females (*n* = 42)
Endurance Difference by Category	High Left 12%	High Left 34%
	High Right 12%	High Right 50%
Age Category		
49 yr and under	High Left 22%	High Left 7.3%
50 to 60 yr	High Left 33%	High Left 7.3%
Above 60 yr	High Left 11%	High Left 27%
		(Χ^2^ = 8.323, df = 2, *p* = .0156)
Age Category		
49 yr and under	High Right 22%	High Right 20%
50 to 60 yr	High Right 33%	High Right 12%
Above 60 yr	High Right 11%	High Right 29%

Within females, those who drove four-in-hand tended to have higher peak sitting summed HGS than those driving one or two horses (*p* < 0.0653). HGS in the left hand in the sitting position and summed peak values were higher in those drivers handling four horses (*p* < 0.05, Table 4). There were no differences in male driver peak HGS when number of horses in hand, noting that male participants were only represented in the one-horse and two-horse teams.

**Table 4:** Position specific peak hand grip strength (kg) by number of horses in hand driven by females

	One Horse	Two Horses	Four Horses
Sitting Left Hand	21.4 ± 1.3	20.6 ± 4.4	32.5 ± 3.4*
Sitting Right Hand	23.6 ± 1.5	22.9 ± 4.8	30.3 ± 3.7
Summed Peak	44.9 ± 2.7	43.5 ± 9.0	62.8 ± 7.0*

^*^denotes significance (*p* < 0.05) between number of horses within rows

Additional factors affecting hand and arm performance are summarized in Table 5. A total of 33% of males reported wearing gloves while driving, and males also reported more cold weather impacts on hand fatigue. Conversely, 78% of females wear gloves while driving, and fewer attribute fatigue to cold weather. More females reported experiencing hand fatigue while driving, with 25% attributing fatigue to the marathon phase. Drivers attributed some fatigue during practicing for events, and 16% of females reported diagnosed arthritis in the wrist or hands within the last six months.

**Table 5:** Equestrian-specific variables by sex

	Males (*n* = 9)	Females (*n* = 42)
Fatigue during combined driving events	22%	37%
Rubs or blisters during combined driving events	0%	12%
Cold weather causes hand fatigue	60%	23%
Practice for events causes hand fatigue	20%	46%
One elbow or forearm injured in last 6 months	0%	11%
Both wrists/hands injured within last 6 months	11%	0%
Arthritis in wrists/hands within last 6 months	0%	16%

Pain and injury reported were in the left side for joint and arthritis diagnosis (Table 6). Muscle strain/pain was predominant (80%) on the right side. No differences were realized in reported injury and summed or peak HGS by hand. No relationships between reported hand injury within the last six months and years of driving experience on HGS were identified. Additional injuries reported by females included concussion (32%), lower body including ankle, pelvis, and hip (16%), and spine (including neck, 13%). Males reported spine and shoulder injuries in equal proportion (25%) which occurred in competitive driving.

**Table 6:** Reported injury by side in hands, wrists, forearm, and elbow within 6-months of event

	Right Side	Left Side
Muscle Strain/Pain	80%	20%
Broken Bone	100%	0%
Joint Strain/Pain	43%	57%
Arthritis Diagnosis	33%	67%

Table 7 represents the entire population as there were no differences by sex in HGE measure. To quantify the association of hand dominance and HGE, we assessed differences in the HGE category between dominant and non-dominant hands. Hand endurance measures were significant for all participants when HGE is high on the dominant hand; HGE is also high on the non-dominant hand (Table 7; Χ^2^ = 17.22, df = 1, *p* < .0001). In addition, demographic and equestrian-specific variables were included in the analysis. The same differences were realized by age, with high dominant and high non-dominant HGE associations (Table 7). Similarly, if the HGE was low in the dominant hand within age, the non-dominant HGE was also low. Those participants in the obese BMI categories were more bilaterally balanced in HGE in both the highest (high dominant/high non-dominant) and lowest (low dominant/low non-dominant) HGE (Χ^2^ = 12.393, df = 1, *p* = .0004). Those participants in the normal BMI category did not realize HGE differences between hands. The association of horses in hand demonstrated that the dominant to non-dominant hand HGE category was similar on the two ends of the spectrum: high-high, and low-low. When considering years of driving experience, those drivers with less than five years and over 30 years also followed the high-high and low-low pattern.

**Table 7:** Frequencies of bilateral endurance balance category and equestrian variable associations for all participants

	High Dominant/High Non-Dominant	High Dominant/Low Non-Dominant	Low Dominant/High Non-Dominant	Low Dominant/Low Non-Dominant	
Endurance Category	42%	20%	4%	34%	*Χ2 = 17.22, df = 1, p < .0001*
Age Category					
Below 49 yr (*n* = 18)	28%	28%	0%	44%	*Χ2 = 7.407, df = 1, p = .0065*
50 to 60 yr (*n* = 15)	33%	20%	7%	40%	*Χ2 = 3.86, df = 1, p = .0493*
Above 60 yr (*n* = 17)	65%	12%	6%	18%	*Χ2 = 4.936, df = 1, p = .0263*
BMI Category					
Underweight (*n* = 1)	100%	–	–	–	
Normal (*n* = 19)	32%	32%	11%	26%	Χ2 = 0.853, df = 1, *p* = .3558
Overweight (*n* = 15)	27%	20%	0%	53%	*Χ2 = 7.837, df = 1, p = .0051*
Obese (*n* = 15)	67%	7%	0%	27%	*Χ2 = 12.393, df = 1, p = .0004*
Horses in Hand					
One (*n* = 41)	412%	17%	5%	37%	*Χ2 = 15.33, df = 1, p < .0001*
Two (*n* = 4)	50%	0%	0%	50%	*Χ2 = 5.55, df = 1, p = .0185*
Four (*n* = 5)	40%	60%	–	–	
Driving Experience					
< 5 yrs. (*n* = 15)	47%	6%	0%	47%	*Χ2 = 14.699, df = 1, p = .0001*
6 to 15 yrs. (*n* = 18)	28%	33%	11%	28%	Χ2 = 0.523, df = 1, *p* = .4696
16 to 29 yrs. (*n* = 8)	50%	25%	0%	25%	Χ2 = 3.452, df = 1, *p* = .0632
> 30 yrs. (*n* = 9)	56%	11%	0%	33%	*Χ2 = 6.959, df = 1, p = .0083*
Barn Chores					
< 25% chores (*n* = 6)	67%	17%	0%	17%	Χ2 = 2.634, df = 1, *p* = .1046
26–74% chores (*n* = 8)	75%	0%	0%	25%	*Χ2 = 8.997, df = 1, p = .0027*
> 75% chores (*n* = 36)	31%	25%	6%	39%	*Χ2 = 7.510, df = 1, p = .0061*
Wear Gloves					
Yes (*n* = 43)	49%	17%	6%	29%	*Χ2 = 11.047, df = 1, p = .0009*
No (*n* = 7)	29%	14%	0%	57%	*Χ2 = 4.557, df = 1, p = .0328*
Fatigue Perception					
Yes (*n* = 31)	47%	18%	0%	35%	*Χ2 = 10.617, df = 1, p = .0011*
No (*n* = 19)	47%	21%	5%	26%	*Χ2 = 4.853, df = 1, p*

Engagement in barn activities, activities, and driving sports that require both strength and dexterity of hands, did not differ between sexes. On average, females complete activities 82% of the time, and males 80%. When looking within sex, females over 60 years of age reported an average of 80.8%, ages 50–60 reported 94.5%, and females under 50 reported 74%, trending towards significance. Males over 50 years old reported an average of 68% where males under 50 years old reported completing 95% of barn activities.

When observing frequencies of grip endurance and the equine-specific categories, combined drivers between 50 and 60 and under 50 (*p* = 0.06) tended to be different for females that complete fewer activities to have a greater difference between right and left-hand endurance (e.g. their left hand being weaker than their right). When considering effects of barn activities, dominance, and HGE for all participants, there was greater bilateral balance within the 26–74% chore category (Table 7, Χ^2^ = 8.997, df = 1, *p* = .0027). When completing over 75% of the activities, there was less bilateral balance and less frequency in the high-high category than other groups of barn activities. Glove wearing was also associated with bilateral endurance balance. For those who wear gloves, a higher proportion of endurance category balance between dominant and non-dominant (49% high-high and 29% low-low; Χ2 = 11.047, df = 1, *p* = .0009). Finally, HGE category was impacted by fatigue perception; following previously identified high-high and low-low category differences (Yes, Χ^2^ = 10.617, df = 1, *p* = .0011; No fatigue, Χ^2^ = 4.853, df = 1, *p* = .0279).

## Discussion

We aimed to evaluate combined drivers’ HGS and HGE and compare values to normative data [16]. In addition, we described equestrian-specific variables that qualify aging drivers’ HGS across the age spectrum. We successfully engaged the combined driving community and validated position-specific grip information. We hypothesized that combined drivers’ handgrip strength would be above average normative data based on their respective sex and age range. Based on the results of the current study, this hypothesis was supported in specific age ranges but not for all combined drivers.

We established grip strength and endurance on 42 female and 9 male combined drivers. The ratio of men to women was different than data provided by the American Horse Council, that showed over 90% of equestrians are female outside of the racing industry [12]. With an 82% female to 18% male split, our data showed that more males participate in combined driving than in other recreational and competitive sport. Over two-thirds of the participants were 50 years or older. Having a majority of older participants in this sport allows researchers to evaluate the potential for combined driving to help individuals maintain muscle strength through continuous hand use in an engaging physical activity. Females of all ages remained above the 50th percentile of the reference population (19). Thus our findings support that intentional physical activity maintains muscle strength [11, 17–19].

Position affected HGS measurements. Sitting handgrip with 90-degree elbow flexion measures lower than handgrip in a standing position [20, 21]. Balogun and colleagues attribute this difference to increased cortical and peripheral arousing in muscles, causing continuous sensory feedback between joint receptors, muscles, and the central command while standing when compared to sitting [20]. Additionally, differences are attributed to grip-size, as the standing position was adjusted for an optimal length based on each participant’s index finger. In contrast, the task-specific position was adjusted to mimic driving reins, hence set to the smallest grip setting. This setting is relevant to the driving population as their hands are in a more natural position when set on the smallest grip setting. These findings support previous research comparing grip strength to grip size due to a decrease in cross-bridge attachments formed as the grip size is smaller and below the muscles’ optimal attachment length [22, 23].

Our second aim was to create baseline data on grip endurance in a task-specific position. The methodological approach of a window of 60% ± 10 lbs. at the smallest grip setting was individualized to this study. Although there was testing validity and reliability with holding MVC at 50% as a normalized grip setting, this was harder to replicate in a field test while using a mirror for feedback, as well as in an older population with a decrease in hand steadiness [24]. We allowed for a range of approximately 40–80% of MVC by using a 60% ± 10 window. This larger window provided a more straightforward visual for participants. We established baseline data on endurance and classified results into low and high endurance for comparison of right and left-hand balance of grip differences. Endurance values ranged from 9 to 120 s. To evaluate the relationship between endurance and equestrian specific variables, a binary variable was created where values 0–60 s were coded low(0), and values 61–120 s were coded high based on prior research [1]. Future research should focus on 50% MVC to more directly compare observed values to previous research, while simultaneously providing stratification of the endurance values beyond low and high categories.

We also compared grip strength to normative values by driver age and sex. All drivers who participated in this study were right-hand dominant. Sex differences between older adults is supported in previous research, where as individuals age, the percent of men who “needs improvement” in grip strength steadily increases while the women’s percentage of “needs improvement” remained unchanged [11]. Additionally, there was an increase in women who fell within the “excellent” category while the percentage of men in the “excellent” category remained relatively the same as they aged [11]. The findings of our study align with Rantanen’s findings, as the eldest age category in women remained above the 50th percentile, while men in the same age category fell below the 50th percentile. The continuous use of intrinsic and extrinsic muscles could contribute to combined drivers’ ability to maintain grip strength relative to age and sex. Furthermore, the ability to maintain grip strength in females is attributed to maintaining muscle strength in both the elbow extensors and flexors. On average, women have a smaller percentage of muscle in their arms to begin with and maintain for an extended period, while men have the potential to lose more muscle and strength as they age [25].

Our fourth purpose was to determine associations between equestrian-specific and grip variables. Sixty percent of males reported cold weather causing their hands to fatigue compared to only 23% of females reporting the same. Previous studies show females’ fingertips and hands cool down faster than males [26], suggesting women might experience more fatigue due to cold weather. However, this could be related to only 33.3% of men versus 63.5% of women wearing gloves while driving outside competition conditions. Additionally, 13.7% of the cohort agreed they only wear gloves while driving when it is cold out, and 72% of them were females. Between always wearing gloves and only wearing gloves when cold, only 55.5% of males wear gloves outside of competition, accounting for a larger fatigue rating in cold weather from the male drivers. This study provides a baseline of fatigue perceptions in this community and requires further exploration in in the equestrian community.

Barn activities seem to promote bilateral endurance balance when less than 75% of activities are completed. A dampening effect on balance in endurance from dominant to non-dominant was observed at greater than 75% of barn activities. It was observed that combined drivers within the low-dominant category remained in the low-non-dominant category even when they completed a higher percentage of activities. This finding suggests that activities may promote endurance balance, but if endurance is low, it remains low regardless of barn chore engagement. There was a higher frequency with older females to fall into the bilaterally high endurance groups, which could be associated with older females reporting completing a higher percentage of barn activities. On average, females in the 51–60 and older than 60 groups reported completing a higher percentage of barn activities than females under 50 years old. Our data suggest that caring for horses provides an additive effect on HGS and could be protective as adults’ age.

Our study was limited by the sample size, particularly of male participants. However, we did show a greater number of male participants than previous research recorded of 18% compared to < 10% [12]. Participants were encouraged verbally throughout both MVC and endurance testing to mimic the competitive experience. Combined drivers did express concern that the endurance test might influence their driving performance. Such concerns could be a psychological factor and may explain ranges of values (e.g. 22–120 s on the dominant hand and 9–120 s on the non-dominant hand). This range is comparable to prior validity and reliability of grip endurance tests and how endurance relies on physiological and psychological factors, including motivation [17, 27]. Additional research considering the motivation and psychological factors of collecting data at a live event are necessary for this community. Future research should evaluate other measurement methods with equipment more similar to reins, as some participants mentioned that the manual dynamometer was uncomfortable to hold, and was not representative of how reins feel during driving activity. Additionally, adding dexterity tests and creating a rein board with force transducers are recommended next steps. Finally, though drivers are the only discipline that use hands as the main communication aid to the horse(s), similar research in all equestrian disciplines are suggested. Understanding how different disciplines maintain strength throughout their lifetime as an equestrian athlete is vital to understand the lifelong benefits of equestrian sport.

## Conclusion

This study demonstrates the exceptional ability of senior-level athletes to perform in driving equestrian activities. Driving more horses in a team develops strength and endurance and increased years of experience provides protective bilateral balance of grip Furthermore, hand dominance did not affect performance. Females over 60 years old have greater endurance values than younger drivers, specifically in the left hand, even though all participants were right-hand dominant. This finding suggests that combined driving could be a beneficial way to maintain higher levels of fitness and strength in older females and may improve other aging outcomes.

## Supplementary Information

**Additional file 1.**

## Data Availability

The datasets used and analyzed during the current study are available from the corresponding author on reasonable request.

## Abbreviations

CDE: Combined Driving Event
QoL: Quality of Life
MVC: Maximal Voluntary Control
HGE: Handgrip Endurance
HGS: Handgrip Strength

## References

[1] Syddall HE, Westbury LD, Dodds R, Dennison E, Cooper C, Sayer AA (2017). Mortality in the Hertfordshire ageing study: association with level and loss of hand grip strength in later life. Age Ageing.

[2] Iconaru EI, Ciucurel MM, Georgescu L, Ciucurel C (2018). Hand grip strength as a physical biomarker of aging from the perspective of a Fibonacci mathematical modeling. BMC Geriatr.

[3] Lee SC, Wu LC, Chiang SL, Lu LH, Chen CY, Lin CH, Ni CH, Lin CH (2020). Validating the capability for measuring age-related changes in grip-force strength using a digital hand-held dynamometer in healthy young and elderly adults. Biomed Res Int.

[4] Lee JA, Sechachalam S (2016). The effect of wrist position on grip endurance and grip strength. The Journal of hand surgery.

[5] Haider S, Luger E, Kapan A, Titze S, Lackinger C, Schindler KE, Dorner TE (2016). Associations between daily physical activity, handgrip strength, muscle mass, physical performance and quality of life in prefrail and frail community-dwelling older adults. Qual Life Res.

[6] Marques LP, Confortin SC, Ono LM, Barbosa AR, d’Orsi E (2019). Quality of life associated with handgrip strength and sarcopenia: EpiFloripa aging study. Arch Gerontol Geriatr.

[7] Vaz-Patto M, Bueno B, Ribeiro Ó, Teixeira L, Afonso RM (2017). Association between handgrip strength, walking, age-related illnesses and cognitive status in a sample of Portuguese centenarians. Eur Rev Aging Phys Act.

[8] Grant S, Hynes V, Whittaker A, Aitchison T (1996). Anthropometric, strength, endurance and flexibility characteristics of elite and recreational climbers. J Sports Sci.

[9] Kramer AM, Knudson DV (1992). Grip strength and fatigue in junior college tennis players. Percept Mot Skills.

[10] López-Rivera E, González-Badillo JJ (2019). Comparison of the effects of three hangboard strength and endurance training programs on grip endurance in sport climbers. J Human Kinet.

[11] Rantanen T (2003). Muscle strength, disability and mortality. Scand J Med Sci Sports.

[12] American Horse Council. Economic Impact Study of the US Horse Industry. American Horse Council; 2018.

[13] EUP. Horse Riding Situation in Europe. Barcelona: Competitiveness and Innovation Framework Programme of the European Union; 2014.

[14] Hitchens P, Blizzard L, Jones G, Day L, Fell J (2011). Are physiological attributes of jockeys predictors of falls? A pilot study. BMJ Open.

[15] Hobbs SJ, Baxter J, Broom L, Rossell LA, Sinclair J, Clayton HM (2014). Posture, flexibility and grip strength in horse riders. J Human Kinet.

[16] Perna FM, Coa K, Troiano RP, Lawman HG, Wang CY, Li Y, Moser RP, Ciccolo JT, Comstock BA, Kraemer WJ (2016). Muscular grip strength estimates of the US population from the national health and nutrition examination survey 2011–2012. J Strength Condition Res.

[17] Capodaglio P, Maestri R, Bazzini G (1997). Reliability of a hand gripping endurance test. Ergonomics..

[18] Rantanen T, Guralnik JM, Foley D, Masaki K, Leveille S, Curb JD, White L (1999). Midlife hand grip strength as a predictor of old age disability. Jama..

[19] Taekema DG, Gussekloo J, Maier AB, Westendorp RG, de Craen AJ (2010). Handgrip strength as a predictor of functional, psychological and social health. A prospective population-based study among the oldest old. Age Ageing.

[20] Balogun JA, Akomolafe CT, Amusa LO (1991). Grip strength: effects of testing posture and elbow position. Arch Phys Med Rehabil.

[21] El-Sais WM, Mohammad WS. Influence of different testing postures on hand grip strength. Eur Sci J. 2014;10(36).

[22] Barut C, Demirel P (2012). Influence of testing posture and elbow position on grip strength. Med J Islamic World Acad Sci.

[23] Blackwell JR, Kornatz KW, Heath EM (1999). Effect of grip span on maximal grip force and fatigue of flexor digitorum superficialis. Appl Ergon.

[24] Blomkvist AW, Eika F, de Bruin ED, Andersen S, Jorgensen M (2018). Handgrip force steadiness in young and older adults: a reproducibility study. BMC Musculoskelet Disord.

[25] Janssen I, Heymsfield SB, Wang Z, Ross R (2000). Skeletal muscle mass and distribution in 468 men and women aged 18–88 yr. J Appl Physiol.

[26] Jay O, Havenith G (2004). Finger skin cooling on contact with cold materials: an investigation of male and female responses during short-term exposures with a view on hand and finger size. Eur J Appl Physiol.

[27] Reuter SE, Massy-Westropp N, Evans AM (2011). Reliability and validity of indices of hand-grip strength and endurance. Aust Occup Ther J.

