# Supplementary material for: Combined driving: task-specific position impacts grip strength of equestrian athletes
Source: Eur Rev Aging Phys Act. 2022 Jan 10;19:2. doi: 10.1186/s11556-021-00282-w (PMC8903501; doi:10.1186/s11556-021-00282-w)
Supplement: Supplementary file 1 — Additional file 1. [file 11556_2021_282_MOESM1_ESM.pdf]

This questionnaire is one component of the competitive driver performance testing and includes questions about exercise and strength training practices, and self-reporting of prior injury. These data will be utilized in conjunction with physical performance data to more thoroughly characterize athletic performance.

Are you at least 18 years of age?

a. Yes
b. No (if no, send to end of survey)

How old are you?

a. 18-25 years
b. 25-30 years
c. 31-40 years
d. 41-50 years
e. 51-60 years
f. Over 60 years
g. Prefer not to answer

What is your ethnic origin?

a. White or Caucasian
b. Hispanic/Latino
c. Black or African American
d. American Indian of Alaska Native
e. Asian
f. Native Hawaiian or Pacific Islander
g. Other (Specify)
h. Prefer not to answer

How much do you weight?

a. Less than 100 lbs/ 45 kilos
b. 100-120 lbs/45-54 kilos
c. 121-140 lbs/55-63 kilos
d. 141-160 lbs/64-72 kilos
e. 161-180 lbs/73-81 kilos
f. 181-200 lbs/82-90 kilos
g. 201-220 lbs/83-99 kilos
h. More than 22o lbs / more than 100 kilos
i. Prefer not to answer

How tall are you?

a. Under 5’ / 154 cm
b. 5’1” – 5’4” / 154-163 cm
c. 5’5” – 5’8” / 164-173 cm
d. 5’9” – 6’2” / 174 – 190 cm

e. Over 6’2” / 190 cm
f. Prefer not to answer

At what level of combined driving do you currently participate (if participating in multiple levels, choose the highest level)?

a. Preliminary
b. Advanced
c. Intermediate
d. FEI 1 Star
e. FEI 2 Star

How many horses do you drive at a time during competition (if different numbers choose the most horses you compete with at a single time)?

a. Single (1)
b. Pair (2)
c. Four-in-hand (4)

How long have you been participating in combined driving?

a. Just started (less than 1 year)
b. 1-3 years
c. 4-6 years
d. 7-10 years
e. 11-20 years
f. 21-30 years
g. Over 30 years

What percent of bar work (i.e., feeding, mucking stalls, etc.), and prepping the horse(s) (i.e., grooming, harnessing, hooking) do you partake in on a regular basis when not competing?

a. Less than 10%
b. 11-25%
c. 26-50%
d. 51-75%
e. Over 75%

Please rate your level of agreement for each of the following statements on a three point scale (Agree, Neutral, Disagree):

a. I wear gloves while driving outside of competition
b. I wear gloves only when the temperature is cold
c. My hands fatigue while driving
d. I get blisters or rubs on my hands from driving

If your hands fatigue when driving, when do they fatigue? (Select all that apply)

a. While practicing
b. While competing
c. During inclement weather
d. Other (specify)

If you hands fatigue when competing, during which phase(s) do they fatigue most? (Select all that apply)

a. Dressage
b. Marathon
c. Cones

Select your body part(s) that have been injured as a result of competitive driving.

a. Face (nose, mouth, eyes, jaw, etc.)
b. Neck (above shoulders)
c. Collar Bone
d. Chest/ Rib Cage
e. Spine – Upper back (Shoulders to end of ribs)
f. Spine – Lower back (From bottom of ribs to top of pelvis)
g. Shoulder/Upper Arm
h. Lower arm (elbow, forearm, wrist)
i. Hands
j. Hips and Pelvis
k. Upper leg (from hips to knee)
l. Knee
m. Lower leg (from knee to ankle)
n. Ankle
o. Feet

For the areas selected above, what was the type of injury you experienced?

a. Muscle Strain/ Pain
b. Joint Strain/ Pain
c. Broken Bone

Have you injured, or do you experience any pain in your hands, wrists, forearms, or elbows during the past 6 months?

a. Yes
b. No

If yes, where are you experiencing pain or where were you injured? (select all that apply)

a. Left hand
b. Left wrist
c. Left forearm
d. Left elbow
e. Right hand
f. Right wrist
g. Right forearm
h. Right elbow

For each of the areas selected above, what kind of injury or pain do you experience? (select all that apply)

a. Muscle strain/ pain
b. Broken bone
c. Joint strain/ pain
d. Crushed/ jammed
e. Diagnosed arthritis
f. Pulled/ drug

We value community-based participation in optimizing research and performance for all equestrians. Your participation in this survey will aid us in developing next steps for enhancing understanding of safe practices and performance for all competitive drivers. Thank you!
